# A Homozygous 1.16 Megabases Microdeletion at 8p22 Including The Whole *TUSC3* in A Three Years Old Girl with Intellectual Disability and Speech Delay 

**DOI:** 10.22074/cellj.2020.6606

**Published:** 2019-09-08

**Authors:** Evren Gumus

**Affiliations:** 1 Department of Medical Genetics, Faculty of Medicine, University of Harran, Sanliurfa, Turkey; 2 Department of Medical Genetics, Faculty of Medicine, University of Mugla Sitki Kocman, Mugla, Turkey

**Keywords:** Deletion, Intellectual Disability, Microarray, TUSC3

## Abstract

Intellectual disability (ID) is defined as an intelligence quotient (IQ) level below than 70. In the present paper, a 1.16 megabases (Mb) homozygous deletion in the 8p22 region was identified in a three years old girl with ID, speech and developmental delays. This is the first report from Turkey with this form of ID. The present paper demonstrates that application of microarray technique to help clinicians, especially when clinical diagnosis includes a complex group of disorders (such as ID) and differential diagnostic list is broad.

## Introduction

Intellectual disability (ID) is an important indicator in the differential diagnosis of genetic diseases. ID comprises two types: syndromic ID (S-ID) and nonsyndromic ID (NS-ID). In S-ID, individuals show one/variousmajor dysmorphic finding(s) or co-morbidities in addition to ID. In NS-ID, an isolated ID is observed. However, it is not always possible to make a clear distinction between these two forms (1)also referred to as mental retardation (MR. According to the inheritance pattern, it is possible to distinguish NS-ID into three main groups: non-syndromic X-linked ID (NS-XLID), non-syndromic autosomal dominant ID (NS-ADID) and non-syndromic autosomal recessive ID (NS-ARID). Regarding NS-ARID, up to 2004, four genes had been identified and up to 2011 they extended to eight genes. Currently, about 40 genes have been recognized corresponding to this group of abnormalities. One of these genes is the Tumor Suppressor Candidate 3 (*TUSC3*) gene. *TUSC3* plays a key role in the N-linked glycosylation process, which is located at the p arm of chromosome 8, containing 11 exons and it is approximately 0.22 megabase (Mb) length. The *TUSC3* gene-associated NS-ARID has been described to date in less than 10 families and less than 30 patients (2-4). 

## Case Report

The study was performed in accordance with the Declaration of Helsinki 2013, the principles of Good Clinical Practice and Local Ethic Regulation (code: 28617). Informed consent was obtained for genetic analysis of the patient, the publication of patient data and photos. 

**Fig.1:**
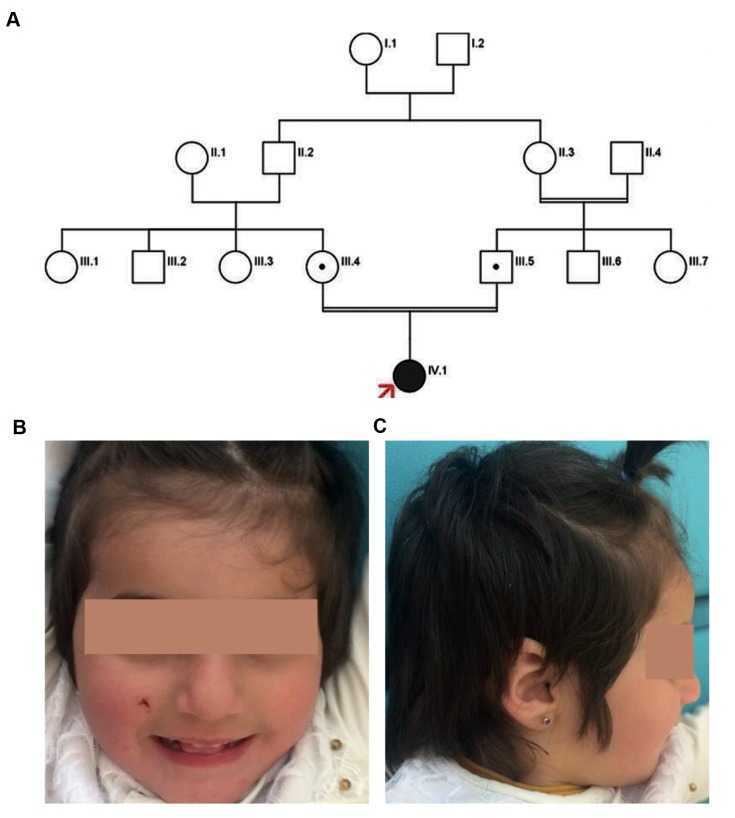
The patient’s pedigree and observed dysmorphic features are indicated. A. The pedigree, B, and C. Front and lateral views of the patient at age 2 years and 8 months shows epicanthus, broad nasal base and thin upper lip.

A three years old child born to healthy 21-years-old mother and 23-years-old father with Turkish origin. The parents are first degree cousins (Fig .1A). Her birth weight was 2950 g (10-25 percentile) and birth length was 49 cm (25-50 percentile). Occipitofrontal circumference (OFC) at birth was not recorded. The patient was referred to our department with intellectual disability, developmental delay, speech delay and minor dysmorphic features. On the examination, her weight, length and OFC is respectively 11.7 kg (3-10 percentile), 96 cm (50-75 percentile) and 47 cm (10-25 percentile). She sat at 22 months, while she is not yet able to walk independently and her speech skills had not been developed. Minor dysmorphic features, such as epicanthus, broad nasal base and thin upper lipare were noted (Fig .1B, C). The creatine kinase level of the patient was 98 U/L (N: 22-198 U/L). Copy number variation of *SMN1* gene is normal. ID was estimated as severe to profound. 

**Fig.2:**
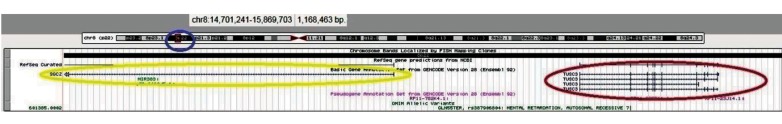
Homozygous deletion of 8p22 region, composed of the whole *TUSC3, miR-383* and a portion of *SGCZ* gene (Adapted from *UCSC* Genome Browser).

**Fig.3:**
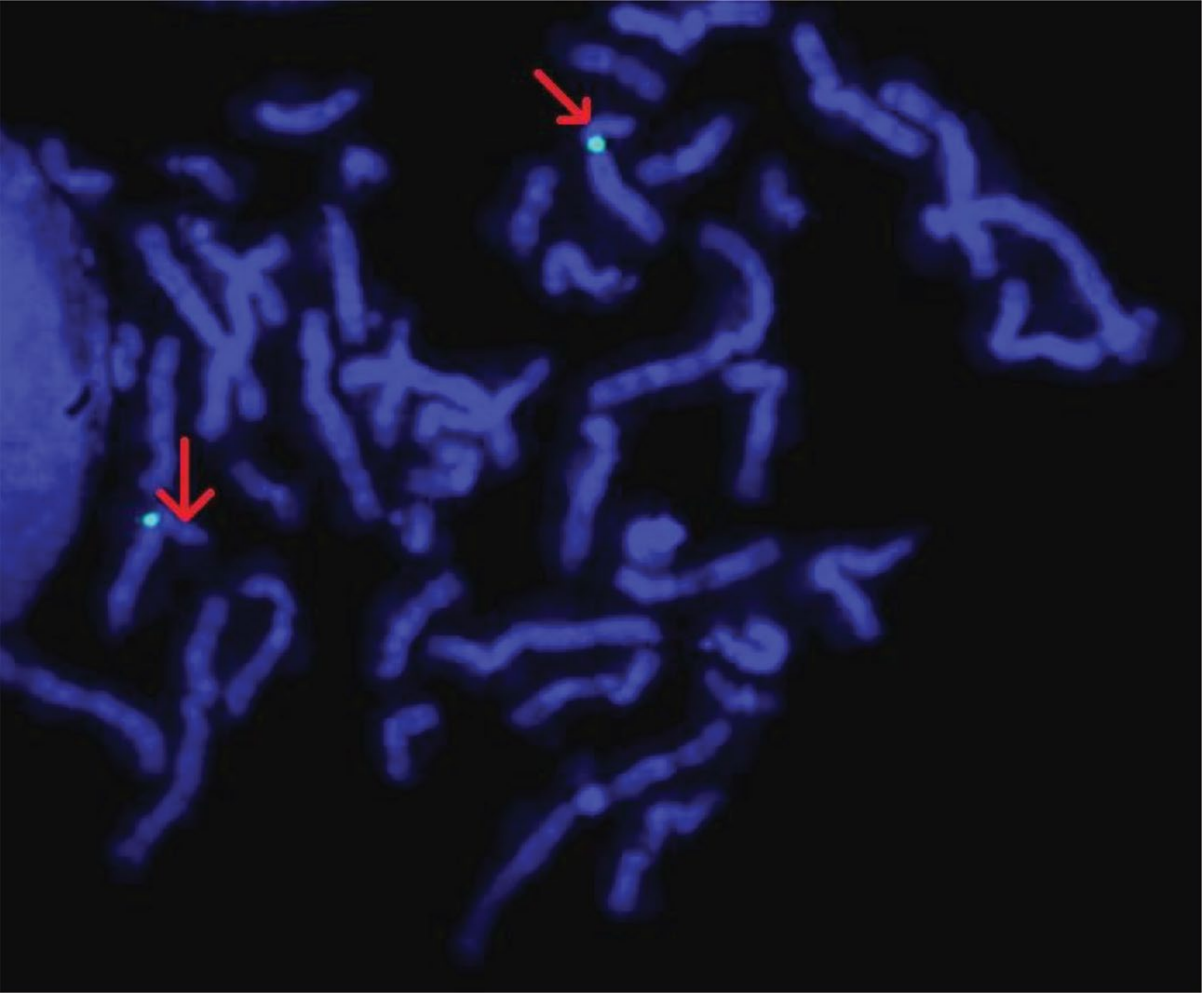
FISH technique analysis was performed by the locus specific probe RP11-165A17, designing to 8p22 and demonstrating no signal (red arrows). A specific probe for the centromeric region of chromosome 8 was utilized as control (green signals).

**Fig.4:**
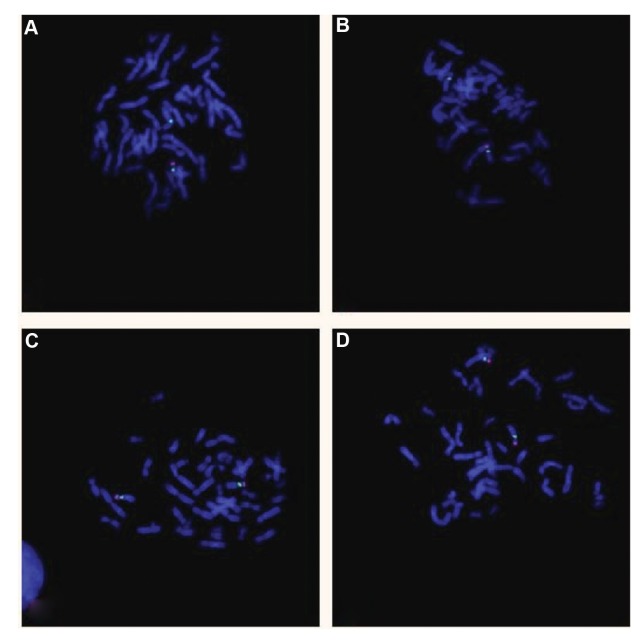
FISH technique analysis was performed with the locus specific probe RP11-165A17, designing to 8p22 (red signals), and a specific probe for the centromeric region of chromosome 8 was utilized as control (green signals). **A.** Mother, **B.** Father, **C.** Healthy woman, and **D.** Healthy man.

Electroencephalography (EEG), electromyography (EMG) and brain magnetic resonance imaging (MRI) were normal. Ophthalmological examination revealed strabismus. Conventional karyotype analysis shows normal female: 46, XX. Chromosomal microarray studies were performed with the Affymetrix CytoScan Optima (315k; Thermo Fisher Scientific, USA) chips from the DNA obtained from her peripheral blood. All data were analyzed in the ChAS 3.1 program (Thermo Fisher Scientific). Microarray result showed a 1.16 Mb homozygous deletion, namelyarr[hg19] 8p22(14,701,24115,869,703) x0. This region contains the whole TUSC3, miR-383 and a portion of the sarcoglycan, zeta (SGCZ) gene (Fig .2). Genetic analysis of her mother and father showed arr[hg19] 8p22 (14,701,241-15,869,703) x1, indicating both parents are heterozygous for this deletion. For confirmation of this deletion in the index patient, fluorescence in situ hybridization (FISH) analysis was performed with the locus specific probe RP11-165A17 (Empire Genomics LLC, USA), mapping to 8p22, and for the control Vysis CEP 8 (D8Z2) Spectrum Green Probe (Abbott Molecular, USA) (Fig .3). These findings also validated the heterozygous deletion in the individual parents. No similar deletion was observed in any healthy woman or man originated from the local population (Fig .4). 

## Discussion

In the present paper, a 1.16 Mb homozygous deletion within the 8p22 region was identified in a three years old girl with ID, speech and developmental delays. This is the first report from Turkey, presenting this form of ID.

microRNAs are small (containing about 22 nucleotides) and extremely preserved non-coding RNA molecules involved in the arrangement of gene expression (5). There is no evidence that *miR-383* plays role in neurodevelopmental processes, but it has been shown to play a role in the etiopathogenesis of ovarian, stomach, colon, prostate and thyroid cancers (6). No malignancy was identified by Piovani et al. (4) in the patient with an 8p22 deletion including *TUSC3*, *SGCZ* and *miR-383* regions. Although at the time of assessment, there is no evidence of malignancy in our patient, we recommend that this case should be followed-up in later stages of her life. Since this effect is only observed in the homozygous case, other family members do not need to be included in the follow-up program (2).

Mutations in β, γ, δ and α sarcoglycans have been associated with limb-girdle muscular dystrophy, myoclonic dystonia and dilated cardiomyopathy, but no phenotype relation has yet been reported for *SGCZ* (7) in a proportion of cases, by mutations of the maternally imprinted epsilon-sarcoglycan gene (SGCE. This has also been implicated by Piovani et al. (4). 

The present paper is the first case with deletion of whole TUSC3 gene. Mouse studies have shown that Tusc3 protein is related to synaptic plasticity, learning continuum and memory (8, 9). There are publications reporting that this interaction is performed via magnesium membrane transport system (10). Reduction of TUSC3 functional expression in zebrafish embryos results in the early developmental arrest (9). The underlying mechanism of ID associated with the *TUSC3* is mainly attributed to this phenomenon. While low TUSC3 protein expression levels are observed in the adult brain, high expression levels are detected in the fetal brain and cerebellum (2). This effect is manifested by ID and developmental delay, observing at an early age. Copy number and single nucleotide variations in the *TUSC3* have been associated with the autosomal recessive syndromic or non-syndromic ID. Previous clinical studies have presented moderate to severe ID, as a common feature in all of these patients. Additionally, speech problems, minor dysmorphic features and developmental delay was also observed in most of these patients (Table 1). Congenital anomalies, including syndactyly and undescended testis, were also found in less than five patients. Similar to our patient, no congenital anomaly was observed in the other patients (3, 4, 8, 11, 12). Although many of these patients are non-syndromic, presence of the syndromic features in a few patients is an indication that this distinction is not certain in autosomal recessive ID. 

**Table 1:** Major clinical features of the previously reported patients and the present case


Findings	Garshasbi et al. (8)	Loddo et al. (3)	El Chehadeh et al. (11)	Piovani et al. (4)	Al-Amri et al. (12)	Present case

Number of patients	7	1	2	1	4	1
Intellectual disability	+	+	+	+	+	+
Speech delay	+	+	+	+	+	+
Microcephaly	1/7	-	-	NA	3/4	-
Consanguinity	Yes	No	Yes	No	Yes	Yes
Height (length)	NA	50p	50p	50p	3-10p	50-75p
Weight	NA	25-50p	50p	50p	3-50p	3-10p
Sat with no support	NA	18 M	15 M	Delayed	NA	22 M
Independent walking	NA	2 Y	2 Y	Delayed	19 M	Not yet
Speech	NA	Delayed	Delayed	Delayed	Delayed	No speech


NA; Not available, M; Month, Y; Year, and P; Percentile.

## Conclusion

 Current paper indicate the extensive implementation of microarray technique to assist clinicians, particularly in the case of complex disease groups and wide list of differential diagnosis. 
